# The prevalence of Mycoplasma hominis in Outpatients at a Tertiary Care Hospital in East India

**DOI:** 10.7759/cureus.31110

**Published:** 2022-11-04

**Authors:** Jasoda Majhi, Dharitri Mohapatra, Nirupama Chayani

**Affiliations:** 1 Microbiology, District Headquarter Hospital, Nuapada, Nuapada, IND; 2 Medical Microbiology, Shri Jagannath Medical College and Hospital, Puri, IND; 3 Medical Microbiology, Srirama Chandra Bhanja (SCB) Medical College and Hospital, Cuttack, IND

**Keywords:** prevalence, polymerase chain reaction, mycoplasma, genital mycoplasma, culture

## Abstract

Introduction

*Mycoplasma hominis (M. hominis) *is the first Mycoplasma isolated from humans in the year 1937. Though regarded as a commensal of the urogenital tract, it has been implicated in various genital and extra-genital infections namely bacterial vaginosis, cervicitis, pelvic inflammatory diseases, pyelonephritis, premature rupture of the membrane in pregnancy, infertility, sepsis in newborns, etc. The pathogenesis, prevalence, and epidemiology of genital mycoplasmas in general and *M. hominis *in particular in Indian women have been studied very minimally. This study aimed to study the prevalence of *M. hominis* carriage among symptomatic and asymptomatic sexually active women attending to the outpatient department of a tertiary care hospital in East India with or without clinically suspected genitourinary infections and to compare the detection of *M. hominis* by polymerase chain reaction (PCR) as compared to that of culture.

Methods

In this observational study, sterile Dacron swabs were used to collect two samples each from the genitourinary tract of 110 sexually active women aged 15-45 years (80 cases and 30 control). One sample was inoculated in mycoplasma broth for culture. The other was used for PCR to detect the presence of the *M. hominis* gene.

Results

Culture positivity for mycoplasma was seen in 4/80 (5%) patients clinically suspected of genitourinary infection (cases) based on their presenting signs and symptoms. In those without such suspicion (control), all cultures were negative (p=0.021). PCR was positive for *M. hominis* in 22 (20%) samples. Considering the PCR as the gold standard the sensitivity, specificity, positive predictive value (PPV), and negative predictive value (NPV) of culture are found to be 18.18%, 100%, 100%, and 88.25% respectively. The highest prevalence of *M. hominis* was in the age group 20-24 years (9/24) and 50% of all detections (11/22) were among 25-29 years. Detections were more frequent among patients with multiparity, multiple sexual partners, intrauterine contraceptive devices, lower socioeconomic status, and lower educational status.

Conclusion

Our study results showed that the presence of *M. hominis* is significantly higher in cases than in the control group. The study also indicates the need for continued research on this bacterium both in patients with genital symptoms and in asymptomatic patients.

## Introduction

*Mycoplasma hominis (M. hominis)* is the first Mycoplasma isolated from humans in the year 1937 [1]. Urogenital mycoplasma, such as *M. hominis,* are common commensals in the urogenital tract of sexually active healthy adults [2]. However, it is also suspected to be a causative agent of various diseases like bacterial vaginosis, pelvic inflammatory diseases (PID), pyelonephritis, post-partum and post-abortion fever, and premature rupture of membranes [3-5]. The transmission is believed to be acquired sexually. These bacteria usually affect young people of reproductive age, of low socioeconomic status, are sexually active, and with multiple partners [6].

The prevalence of genital Mycoplasma in general and *M. hominis *in particular in Indian women have been explored minimally. A review of available literature showed the prevalence of *M. hominis *infection contributes to 14% of urogenital infections [7]. No data exist regarding the prevalence in this part of the country to the best of our knowledge. Although screening for conventional STIs (*Neisseria gonorrhoeae* and *Treponema pallidum*) is a routine practice in patients attending STD clinics, screening for genital mycoplasmas is generally not done [8]. Treatment of lower genital tract infection in Indian women is based on the WHO‑guided syndromic approach [9]. However, *M. hominis* is not being adequately treated by syndromic case management protocol of urogenital discharge since it is intrinsically resistant to azithromycin. Genitourinary infections with *M. hominis* require treatment with doxycycline [10]. This study was undertaken to investigate the presence of *M. hominis* in the urogenital tract of sexually active females visiting a tertiary care hospital in East India.

## Materials and methods

The study was approved by the Institutional Ethics Committee (SCB Medical College, Cuttack) vide certificate no 417/18.02.2017. This cross-sectional observational study was conducted on a convenience sample of 110 females consecutively attending the obstetrics and gynecology department of a tertiary care hospital and medical college in Odisha, India. The study adhered to the tenets of the Declaration of Helsinki and maintained patient confidentiality.

Fifteen to 45-year-old sexually active females having symptoms of pain abdomen, cervical bleeding, vaginal discharge, or without symptoms and who provided verbal informed consent to participate in the study were included in the study. Patients younger than 15 years and older than 45 years of age and those who had received anti-microbial therapy within two weeks of the sampling were excluded from the studies.

The sample size was calculated by using the formula n = Z^2^P(1-P)/d^2^, where n = Sample size, Z = Z statistic for a level of confidence (1.96 for 95% confidence level), P = Expected prevalence or proportion (14%) and d = Precision (7%) and a sample size of 96 was calculated.

Data were collected from the participants about their age, socioeconomic status, educational status, history of multiple sexual partners, parity, and the methods used for contraception.

They were studied as two separate groups. Group A included patients complaining of one or more of the following symptoms: pain abdomen, vaginal discharge, cervical bleeding or spotting, frequency of urination, or dysuria. Group B consisted of patients who had none of these symptoms.

Two samples were taken from each patient with the use of sterilized swabs by the investigators. The samples were taken either from the urethral meatus, the vagina, or the endocervix based on the presenting symptoms and clinical examination of the patient. A sampling swab was introduced 0.5-2 cm into the urethral meatus and rotated for a few seconds to collect the urethral swab. Before collecting a vaginal swab, a Cusco’s speculum was placed in the vagina. The swab was introduced into the posterior and lateral fornices of the vagina to collect the vaginal sample. In the case of the cervical swab, with the speculum in place, the sterilized swab was introduced 1-2 cm into the endocervical canal to collect the cervical sample. All swabs were transported to the laboratory in SP-4 glucose broth immediately. One swab was used for the culture while the other was preserved at -20⁰C for molecular assay.

Culture of mycoplasma

The first swab from each patient was pressed against the inner wall of the test tube to release the sample and then the swab was discarded. 0.1mL of the specimen from SP-4 broth was inoculated in mycoplasma broth (PPLO broth supplemented with arginine) and incubated aerobically at 37⁰C for 24-72 hours. If the color of the broth changed from amber to red, a loop-full of the broth was inoculated in the PPLO agar supplemented with arginine. The plates were incubated at 37⁰C for two to five days in a candle jar in the presence of 5% CO_2_. The plates were inspected daily for the appearance of any growth and considered negative if no growth was seen at the end of the fifth day.

Molecular methods

*DNA Extraction*:

The tubes containing the second swabs from the patients were centrifuged at 6,000 rpm for 30 minutes. The supernatant fluid was discarded, and the sediment was poured into a 1.5 mL microtube. DNA extraction was done using a DNA extraction kit (High pure polymerase chain reaction [PCR] Template Preparation, Roche, Germany) as per instructions provided by the manufacturer. To avoid disintegration of the DNA, the DNA sample was put in 0.2 mL aliquot microtubes and maintained at -20⁰C until the PCR test was conducted.

PCR Test for M. hominis Detection

The primer sequence was as: Forward: 5′-CAA TGG CTA ATG CCG GAT ACG C-3′ and Reverse: 5′-GGT ACC GTC AGT CTG CAA T-3′ for 16s ribosomal gene of *M. hominis*. The total volume of the PCR reaction was 25 microliters using premade PCR master mix (CinnaGen, Iran). The length of the *M. hominis* PCR product was 687 bp.

**Figure 1:**
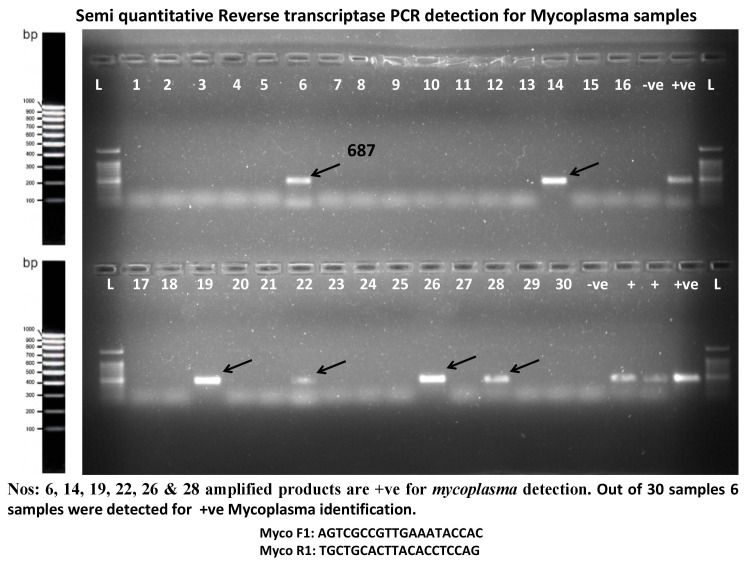
Ethidium bromide strained 2% agar gel shows the PCR amplification product with 16SrRNA gene for Mycoplasma hominis. Lanes 6 and 14 are positive for Mycoplasma hominis with 687 base pair product.

The PCR amplification program was performed in Thermocycler (Eppendorf, Germany). This included initial denaturation at 94⁰C for 10 minutes, followed by 30 cycles of denaturation at 94⁰C for one minute, annealing at 64⁰C for 30 seconds, and extension at 72⁰C for 10 minutes. PCR products were separated by agarose gel electrophoresis in 1.5% gel agarose, stained with ethidium bromide, visualized by ultraviolet light, and photographed (Figure 1). The PCR-positive control was DNA extracted from M. hominis ATCC 15488.

Statistical analysis

All the data were collected and analyzed using Microsoft Excel 2010 (Microsoft Corporation, Washington, DC, United States of America). All results were expressed as absolute frequencies and percentages. A p-value of ≤0.05 was considered statistically significant. The sensitivity, specificity, and positive predictive values were calculated appropriately in comparison to culture and PCR.

## Results

Of the 110 women enrolled, 80 (72.7%) had one or more of the following symptoms: pain abdomen, abnormal vaginal discharge, cervical bleeding or spotting, frequency of urination, or dysuria. They were placed in Group A. Thirty (27.3%) women had no such symptoms and were placed in Group B. Most patients in both groups were in the age group of 20-29 years (62.5% in Group A and 53.3% in Group B; Table 1). *M. hominis* was detected in varying proportions among different age groups of women examined (Table 1). It was mostly seen among young adults. Overall, 50% (11/22) of the detections were in the 25-29 years age group with the highest prevalence (37.5%) in the 20-24 years age group based on the total number of women examined in that age group.

**Table 1:** Demographic profile of patients and detection of genital mycoplasma

Demographic factors	Group A (80) [n (%)]	Group B (30) [n (%)]	Total (110) [n (%)]	Positive for genital mycoplasma [n (%)]	P-value
Age (in years); Range 15-45y					
15-19	2 (2.5)	3 (10)	5 (4.5)	1 (20)	
20-24	20 (25)	4 (13.3)	24 (21.8)	9 (37.5)	
25-29	30 (37.5)	12 (40)	42 (38.2)	11 (20.2)	
30-34	18 (22.5)	8 (26.6)	26 (23.6)	1 (3.8)	
35-39	2 (2.5)	3 (10)	5 (4.5)	0 (0)	
40-45	8 (10)	0 (0)	8 (7.3)	0 (0)	
Parity					
0	2 (2.5)	3 (10)	5 (4.5)	1 (20)	
1	14 (17.5)	5 (16.7)	19 (17.3)	2 (10.5)	
2	28 (35)	10 (33.3)	38 (34.5)	7 (18.4)	
3	36 (45)	12 (40)	48 (43.6)	12 (25)	
Socioeconomic status					
Low	44 (55)	12 (40)	56 (50.9)	14 (25)	0.7
Medium	26 (32.5)	16 (53.3)	42 (38.2)	6 (14.3)	
High	10 (12.5)	2 (6.7)	12 (10.9)	2 (16.7)	
Educational Level					
Nil	5 (6.25)	2 (6.7)	7 (6.4)	1 (14.3)	
Primary school	15 (18.8)	5 (16.6)	20 (18.2)	8 (40)	
Secondary school	20 (25)	3 (10)	23 (20.9)	6 (26.1)	
Above secondary school	40 (50)	20 (66.7)	60 (54.5)	7 (11.7)	
Contraceptive method					
IUD	30 (37.5)	12 (40)	42 (38.2)	12 (28.6)	
OCP	36 (45)	8 (26.7)	44 (40)	5 (11.4)	
Condom	4 (5)	0 (0)	4 (3.6)	0 (0)	
Tubal ligation	8 (10)	7 (23.3)	15 (13.6)	4 (26.7)	
None	2 (2.5)	3 (10)	5 (4.5)	1 (20)	
History of multiple sexual partners					
Present	4 (5)	0 (0)	4 (3.6)	3 (75)	0.04
Absent	76 (95)	30 (100)	106 (96.4)	19 (17.9)	
Clinical presentation					
Symptomatic	80 (100)	-	80 (72.7)	20 (25)	
Cervicitis	44 (55)				
Vaginitis & lower abdominal pain	27 (33.7)				
Urethritis	6 (7.5)				
DUB	3 (3.7)				
Asymptomatic	-	30 (100)	30 (27.3)	2 (6.7)	
Group A = patients with signs and symptoms suggestive of genital infection; Group B = patients with no signs and symptoms suggestive of genital infection; IUD = intrauterine device; OCP= oral contraceptives; DUB = dysfunctional uterine bleeding; n = number of samples					

The commonest presentations in Group A (n=80) were cervicitis (55%) and vaginitis with lower abdominal pain (33.7%). In group B, all 30 samples were culture-negative for mycoplasma. In group A, however, 4/80 (5%) samples showed culture positivity (P=0.021). The results of the semi-quantitative PCR showed a total of that 22/110 (20%) positive cases. Out of the 106 samples that were culture-negative, 18 were positive for *M. hominis* with PCR. 

The isolation rate was 21.2% among those in the low, 14.3% in the medium, and 16.7% in the high socioeconomic groups (Table 1). The two women in Group B found to have *M. hominis* were from the low socioeconomic stratum (P-value = 0.7 using Fisher’s exact test).

Almost all women denied having had multiple sexual partners. Only four women in Group A reported having multiple sexual partners. Three of them were found to be positive for *M. hominis* on a PCR test. The highest prevalence of the infection was found among the women who had primary (40%) or secondary (26%) school-level education. Most of the study participants had studied to a level higher than secondary school (60/110) and the prevalence of the infection was the least (11.7%, 7/60) in this group (Table 1).

Of the 110 patients, 40.0% were on oral contraceptive pills (OCPs) while 38.2% were on intrauterine devices (IUDs) for contraception. In group A, 10 (33%) out of 30 patients using IUDs were positive for *M. hominis* while in group B, two (17%) out of 12 patients using IUDs were positive for *M. hominis* (Table 1).

## Discussion

A total of 22 (20%) out of 110 specimens were found to be positive for *M. hominis* by PCR while four (3.6%) were positive by culture.

Symptomatic and asymptomatic women

We found a statistically significant difference (p= 0.021) in culture positivity for genital mycoplasma among the symptomatic Group A (4/80, 5%) and the asymptomatic Group B (0/30, 0%) patients. There are, however, reports of the prevalence of genital mycoplasma in asymptomatic sexually active women [11]. The long-term reproductive consequences of such colonization in asymptomatic women may need further investigation.

Isolation rate

The prevalence of *M. hominis* among women reportedly ranges between 0.6% and 50.4% [12-15]. Bhatt et al. reported a prevalence of 14.2% for *M. hominis* in women with genital tract infections [16]. A study conducted using multiplex PCR by Dhawan et al. showed the prevalence of *M. hominis* as 14.7%, whereas that conducted by Saigal et al. showed 5.4% for *M. hominis* [8,17]. A recent study from Kerala also showed a prevalence of 10% of *M. hominis *[18]. The prevalence is higher in this study compared to other studies from India, which may be because we took the PCR method as a gold standard.

There is a great variance in the sample population of these studies. Some studies were done exclusively on women from gynecological and sexually transmitted disease (STD) clinics [13], some on pregnant women [5], some on both pregnant and non-pregnant women [15], and some on women living in HIV-endemic areas [12]. Different methods had also been undertaken to detect the prevalence. While some studies used the culture test [15], others used biochemical determinations [13]. Some other studies used PCR [12]. In our study, four (3.6%) of the 110 patients studied showed positivity for genital mycoplasma on culture while 22 (20%) showed PCR positivity for *M. hominis*. 

Age group

The highest prevalence for *M. hominis* was detected in the 20-24 years age group with 50% (11/22) of all detections found in the 25-29 years age group. Only one, out of the 22 detections, was in a woman above 30 years of age. A pattern similar to that in our study has been reported earlier by Njunda et al. [15]. In their study, 16 (24.6%) of the total 65 detections were in the 25-29 years age group while 12/18 (66.7%) women in the 20-24 years age group were detected with genital mycoplasma. Vouga et al [19] also identified young age (p<0.001) as a risk factor for *M. hominis* colonization. Ranjan & Nair also found the 26-35 age group to be highest in isolation of genital *Mycoplasma *[18].

In the studies by Redelinghuys et al. [12], and Agbakoba et al. [20], age distribution did not show any pattern in the isolation of genital mycoplasma. Since transmission of genital mycoplasma is related to sexual activity, sexually active women in any age group can become potential carriers of the organisms.

Parity

*M. hominis* was found among women of varying parity but increased with parity. Overall, 86.4% (19) of the 22 detections were in multiparous women while only one nulliparous and two primiparous women showed the presence of *M. hominis*. One explanation for this disparity may be that multiparous women are likely to be exposed to more sexual contact than nulliparous women. However, in some studies, the majority of nulliparous women also harbor *M. hominis *[21]. In a large study of over 5,000 patients, both univariate and multivariate analyses identified nulliparity (p 0.009) as a risk factor for *Ureaplasma spp*. and/or *M. hominis *colonization during pregnancy [19].

Contraception methods

Only four participants reported the use of condoms for contraception and none of them showed mycoplasma colonization. Consistent condom use is believed to protect against sexual transmission of infections and prevent mycoplasma colonization in women [22,23]. However, Verteramo et al. [24] reported that condom use did not protect against *M. hominis* infection in the women they studied. This suggests a possible nonsexual mode of transmission of the organisms.

Hormonal contraception, such as OCPs, has also been shown to protect against mycoplasma infection [22,25]. In our study, only 5/44 (11%) women on OCPs were found to have *M. hominis*. As in other studies [22,26], a higher prevalence was seen among those who used IUDs (12/42, 29%). The use of copper-containing IUDs rather than levonorgestrel-releasing devices has been associated with nonspecific inflammatory changes that affect the cytology of the cervix and vagina and possibly lead to increased colonization by mycoplasma [26]. We did not collect data about the type of IUDs the women used. The prevalence of mycoplasma among IUD users and those with tubal ligation was similar (29% for IUD versus 27% for tubal ligation). However, we could not find any other study on PubMed about the prevalence of *M. hominis* among women with tubal ligation.

Multiple sexual partners

Although only 4/110 women reported having had multiple sexual partners, all four had symptoms suggestive of urogenital infection like vaginal discharge, pain abdomen, and cervical bleeding, and three showed the presence of *M. hominis*. Women with multiple sexual partners are at higher risk of mycoplasma infections [27,28]. Considering the taboo in the Indian society about women having multiple sexual partners, a biased reporting of sexual history cannot be ruled out.

Socioeconomic groups

Of the 22 women with* M. hominis* infection, 14 (63.6%, Table 1) had low socioeconomic status. Like other STDs, mycoplasma infection is also more common among women of lower socioeconomic strata [29]. However, we must also consider the fact that most of the participants (56/110, 51%) were in the low socioeconomic group and there was not much difference in the prevalence of *M. hominis* among the different socioeconomic groups (Table 1).

Educational status

The prevalence of *M. hominis* decreased with an increase in the educational status of the women. This supports a study by Kalinka et al. [30] that shows that a lower educational status is associated with a higher prevalence of *M. hominis* infection. However, the definition of “primary education” differed from our study where school education was divided into the primary level and secondary level. In contrast to both these studies, Agbakoba et al. [20] found a higher prevalence among women with a higher level of education.

Limitations of the study

This was a single-center study with a limited sample size. We only looked into the *M. hominis* infections and no other genital mycoplasmas. We also did not perform the susceptibility testing which would have given the resistance pattern of the organism in the locality. Socio-demographic factors and epidemiological risk factors were not assessed in detail.

## Conclusions

The study showed a significant detection of *M. hominis* in symptomatic women. It emphasizes the need to include genital mycoplasma surveillance along with other organisms causing sexually transmitted infections. Many men and women may be infected or colonized by mycoplasma while showing no clinical symptoms. Such people can become carriers and spread the microorganism through sexual contact. Adequate sex education must be provided to young, not-too-educated women from the lower socioeconomic sector. They must be taught the importance of reproductive health and avoid multiple sex partners.

This study contributes to the epidemiological data of colonization by *M. hominis* in the geographical region, especially because surveillance of sexually transmitted infections is not robust in India. Further studies are required to better understand the complex role of mycoplasma in the urogenital tract of women. More such studies are needed to know local patterns of prevalence and resistance patterns that will facilitate clinicians to choose the best treatment options for the patients.
